# Peripheral inflammation and neurocognitive impairment: correlations, underlying mechanisms, and therapeutic implications

**DOI:** 10.3389/fnagi.2023.1305790

**Published:** 2023-11-29

**Authors:** Siyou Tan, Wenyan Chen, Gaoyin Kong, Lai Wei, Yubo Xie

**Affiliations:** ^1^Department of Anesthesiology, The First Affiliated Hospital of Guangxi Medical University, Nanning, China; ^2^Department of Anesthesiology, Hunan Provincial People’s Hospital, The First Affiliated Hospital of Hunan Normal University, Changsha, China; ^3^Guangxi Key Laboratory of Enhanced Recovery after Surgery for Gastrointestinal Cancer, The First Affiliated Hospital of Guangxi Medical University, Nanning, China

**Keywords:** peripheral inflammation, neurocognitive impairment, large-scale brain functional network, fMRI, neuroinflammation

## Abstract

Cognitive impairments, such as learning and memory deficits, may occur in susceptible populations including the elderly and patients who are chronically ill or have experienced stressful events, including surgery, infection, and trauma. Accumulating lines of evidence suggested that peripheral inflammation featured by the recruitment of peripheral immune cells and the release of pro-inflammatory cytokines may be activated during aging and these conditions, participating in peripheral immune system-brain communication. Lots of progress has been achieved in deciphering the core bridging mechanism connecting peripheral inflammation and cognitive impairments, which may be helpful in developing early diagnosis, prognosis evaluation, and prevention methods based on peripheral blood circulation system sampling and intervention. In this review, we summarized the evolving evidence on the prevalence of peripheral inflammation-associated neurocognitive impairments and discussed the research advances in the underlying mechanisms. We also highlighted the prevention and treatment strategies against peripheral inflammation-associated cognitive dysfunction.

## 1 Introduction

Accumulating people are suffering from cognitive impairment and dementia globally, whose pathogenic causes may be various, including environmental and genetic. The elderly and those with less brain fragility, such as the previously healthy and young populations, may all suffer from acute neurocognitive deficits post stressful events such as infections, peripheral organ injuries, and non-cranial brain surgery (Fernandez-Castaneda et al., 2022; Suman et al., 2022; Barreto Chang et al., 2023a). This has been observed in both human and animal models, alleviating peripheral inflammation could significantly improve cognition in patients with cognitive impairment. Although the detrimental effect on the brain during these conditions may be attributed to multiple causes, these findings indicated that persistent and dramatic peripheral inflammation may be an important cause of neurocognitive impairment (Bever et al., 2017; Balzano et al., 2020; Wang et al., 2021). Progress has been achieved in the exploration of the distinct pathways between acute or chronic inflammatory activation in the periphery leading to neurocognitive impairment, and in combating neurocognitive impairment based on strategies to counteract peripheral inflammation. In this study, we reviewed the research progress related to peripheral inflammation and neurocognitive dysfunction from the perspectives of the correlation between the level of peripheral inflammation and neurocognitive dysfunction, the brain regions and structures that may be affected by peripheral inflammation, the mechanism of peripheral inflammation-induced central immune-inflammatory activation, and prevention and treatment tools based on inflammatory immunomodulatory strategies.

## 2 Peripheral inflammation and neurocognitive impairment

### 2.1 From peripheral inflammation to neurocognitive impairment

Although peripheral inflammation has been shown to increase the risk of developing cognitive impairment in a growing number of studies, the specific mechanisms by which these inflammation-related processes affect cognitive degradation and when cognitive impairment manifests itself during inflammatory influences are poorly understood.

A recently published Mendelian randomization study (Zhuo et al., 2023) based on a sample of 20,381 cases pointed to a causal relationship between high levels of peripheral inflammation levels and cognitive deterioration, which may be mediated by atrophy of brain volume (gray and white matter) under the influence of inflammation. The study demonstrated that peripheral C-reactive protein (CRP) levels correlated with gray matter volume in the left para hippocampal gyrus (β = −0.05, 95% confidence interval (CI): −0.06 to −0.04, pFDR = 1.07*10^−16^) and cognitive performance (β = −0.03, 95% CI: −0.04 to −0.01, pFDR = 0.001). Since Mendelian randomization is a method of causal inference based on genetic variation that employs genomics-based single nucleotide polymorphism (SNP) as an instrumental variable, this inflammatory trait is carried from the very beginning of life, thus demonstrating cause-to-effect, that is, the detrimental effect of peripheral inflammation on cognition.

Other phenomena explain the role of peripheral inflammation as a cause or predisposing factor for cognitive impairment, such as the severe delirium with significant onset of hallucinations and attentional deficits that we have observed in some cases (e.g., radical prostatectomy and hip arthroplasty patients) during awakening after prolonged or damaging surgeries. This abnormality often occurs despite effective pain control and we hypothesize that it is related to peripheral stress events because postoperative delirium exhibits a strong correlation with surgical category. Despite the fact that most anesthetic drugs act centrally to exert analgesic or sedative effects, the impact of the choice of anesthetic regimen on postoperative neurocognitive function has so far been a difficult question to answer, with several lines of evidence (Sun et al., 2016; Hughes et al., 2021; Bhushan et al., 2022) denying such a correlation in both younger and older populations, but it is undisputed that research on this topic relies on a deeper exploration of the mechanisms of the action of anesthesia drugs and of the formation of cognition.

Anatomical and functional brain imaging evidence has also led us to recognize the potential effects of peripheral inflammation, with the most striking association being that in cognitively normal and even healthy populations, peripheral immune-inflammatory fluctuations have induced alterations in specific brain regions and functional connectivity networks, including the enhancement or weakening of connections in different brain regions, and that such alterations may contribute to the dysregulation of brain functions including behavioral modulation, mood, cognition, or even neuro pathological changes in degenerative diseases (Walker et al., 2020; Oberlin et al., 2021; Arnsten et al., 2023; Ge et al., 2023). This leads us to suggest that peripheral inflammation may precede cognitive impairment. Certainly, the sensitivity and specificity of clinical tools for the identification and diagnosis of neurocognitive impairments confound this link and causality.

Based on accumulating evidence, we believe there is a causal link between peripheral inflammation mediating neurocognitive impairment. However, it is worth noting that this is not a one-way story. The brain may have the capacity to memorize and regulate peripheral immune features. The central nervous system, as the site of “immune memory” formation, is capable of storing immune-related information and monitoring and regulating the state of the peripheral immune system. In Koren et al. study, colitis-associated immune stress induced a significant increase in neuronal activity in the central amygdala, insular cortex, and accessory somatosensory cortex brain regions in mice, and, interestingly, reactivation of neurons in the insular cortex after removal of the immune stress environment recapitulated the activation of immune cells (e.g., γδ, CD8+, and CD4+ T cells, as well as dendritic cells (DC) cells) in the colon and the elevation of cytokine levels, which led to the infiltration of immune cells into the colonic tissues (Koren et al., 2021). In addition, activation of the dorsal motor nucleus of the vagus nerve (DMV) is able to inhibit splenic tumor necrosis factor (TNF) expression and hepatic macrophage infiltration through downstream vagal actions (Kressel et al., 2020; Hur et al., 2023). These studies suggest that neuronal cells in specific regions of the brain have “immune memory” and “immunomodulatory” functions and are capable of regulating the peripheral inflammatory immune environment.

Overall, neurocognitive impairment caused by peripheral inflammation is a complex process of bidirectional interactions and continuous progression. Early intervention and correction of the imbalance between peripheral and central inflammation in at-risk populations to avoid the injurious effects of peripheral inflammation before neurocognitive impairment occurs appears to be of great significance. Effective diagnostic and preventive as well as prognostic assessment tools rely on in-depth studies of the mechanisms linking peripheral inflammation to neurocognition.

### 2.2 Surgery

Perioperative neurocognitive disorders (PND), as a common perioperative complication, can be categorized into preoperatively diagnosed cognitive decline, acute postoperative delirium (POD), delayed postoperative neurocognitive recovery, and postoperative neurocognitive deficits depending on the time of onset, which can lead to a poor prognosis, prolonged hospital stays, reduced quality of life, social dependence, and even death (Kong et al., 2022). PND can occur in up to 65% of elderly patients undergoing surgery and anesthesia (Rudolph and Marcantonio, 2011). Surgery, as a complex combination of multiple stressful events, results in tissue or organ damage and pain, and is often accompanied by an intense inflammatory process. Surgical grade or complexity may also be associated with cognitive deterioration, as higher-graded or complex surgeries tend to cause more severe tissue and organ structural damage and postoperative pain, such as spinal (Barreto Chang et al., 2023b) and cardiac surgeries (Au et al., 2023). In a prospective, multicenter, observational cohort study, 38 (16%) of 246 (≥65 years old) elderly patients undergoing elective surgery developed POD within seven days after surgery, and this group of patients developed functional brain network disorders characterized by decreased strength of functional brain connectivity as assessed by resting-state functional magnetic resonance imaging (rs-fMRI), suggesting that decreased strength of brain connectivity is significantly associated with increased pain after major surgery (Ditzel et al., 2023). Cardiac and major vascular surgery has previously been recognized as a high-risk factor for postoperative cognitive deterioration, as such procedures are often accompanied by significant intraoperative hemodynamic fluctuations, including insufficient cerebral perfusion and tissue oxygenation due to extracorporeal diversion and left subclavian and cephalad trunk artery clamping, as well as intense incisional pain (Sadlonova et al., 2022; de la Varga-Martinez et al., 2023). A retrospective analysis of 71 patients aged 46–64 years undergoing elective cardiac surgery revealed that the incidence of postoperative cognitive dysfunction (POCD) could be as high as 33% (Oyoshi et al., 2023). Elderly female patients undergoing non-cardiac surgery of the peripheral system, such as urogynecological surgery including incontinence surgery, vaginal suspension, vaginal hysterectomy, or laparoscopic vaginal sacral fixation, demonstrated an increased incidence of abnormal cognition at 6 weeks postoperatively (Brandner et al., 2018). Total hip arthroplasty or femoral head replacement can significantly increase the incidence of neurocognitive impairment during the awakening period and while in the hospital (Bhushan et al., 2022), and it is noteworthy that anesthetic drug overuse and general anesthesia are frequently avoided based on a cerebral protection standpoint because patients are often elderly, and a Meta-analysis incorporating eight randomized controlled trials (RCTs) including 3,555 patients reported that there was no significant difference in the incidence of POCD after choosing general or regional anesthesia for elderly patients undergoing such procedures, suggesting that severe peripheral injury is the main cause of cognitive impairment (Bhushan et al., 2022). In addition, the presence of preoperative cognitive impairment for either cardiac or noncardiac surgery can burden the risk of acute neurocognitive impairment after surgery. A meta-analysis of 16 studies comprising 62,179 patients found that patients with preoperative cognitive impairment had a significantly increased risk of acute neurocognitive impairment, i.e., delirium, after cardiac surgery (odds ratio (OR): 8.35; 95% CI: 4.25–16.38; *p* < 0.00001) (Au et al., 2023). In a recent study, among 1,497 patients aged ≥55 years undergoing elective noncardiac surgery (e.g., open/orthopedic, abdominal, or minimally invasive), patients with preoperative cognitive impairment experienced higher calculated time-weighted average (TWA) pain scores and a significantly higher incidence of delirium in the first five days after surgery compared with those with normal preoperative cognition (Ma et al., 2023).

### 2.3 Infection

Infections are recognized risk factors for dementia, including Alzheimer’s disease (AD), vascular dementia, and other types of dementia (Sipila et al., 2021). A variety of pathogens such as herpes simplex virus (HSV) (Lopatko Lindman et al., 2021), human cytomegalovirus (CMV) (Lopatko Lindman et al., 2021), Epstein–Barr virus (EBV) (Zhang N. et al., 2021), severe acute respiratory syndrome coronavirus 2 (SARS-CoV-2) (Fernandez-Castaneda et al., 2022); *Helicobacter pylori* (H.P) (Wang J. et al., 2023), *Treponema pallidum* (TP) (Miklossy, 2015), *Borrelia burgdorferi* (Herrera-Landero et al., 2019), Chlamydia (Lopatko Lindman et al., 2021; Chacko et al., 2022), and Toxoplasma gondii (Torres et al., 2018), may contribute to the cognitive decline. Most of these pathogens can cross the blood–brain barrier (BBB) and involve the meninges or parenchyma, inducing infectious encephalopathies. Recent studies have demonstrated that mild coronavirus disease 2019 (COVID-19) inflammation/infection confined to the lungs that does not involve the brain can lead to significant neuroinflammation and long-term cognitive impairment, pointing to the possibility that the infection/inflammation does not need to occur directly within the brain to cause long-term damage to neurocognitive function (Fernandez-Castaneda et al., 2022). Several cross-sectional studies have pointed out the correlation between H.P. and impaired cognitive function (Cardenas et al., 2019; Zilli et al., 2021). In a recent report from the Framingham Heart Research Center, antibody levels in the serum of H.P-infected individuals were found to be associated with poorer overall cognitive performance (Zilli et al., 2021). Although the mechanisms of H.P-associated cognitive impairment may be multifactorial, further studies have shown that infection-associated chronic inflammation is a major cause of cognitive impairment (Xu et al., 2016; Bairamian et al., 2022; Gonzalez et al., 2023). Notably, pathogenic infections or systemic alterations in the response properties of the body’s immune inflammation promote the cognitive impairment process. In a 5-year follow-up study, CMV infection was associated with more rapid cognitive decline and the development of AD (Bu et al., 2015), while peripheral blood mononuclear cells (PBMCs) in CMV-infected AD patients were more reactive after stimulation than in non-infected patients, resulting in activation of peripheral-central inflammation initiation and expansion (Westman et al., 2014).

### 2.4 Systemic inflammation in chronic diseases

Elderly or chronically ill populations are often characterized by long-term mild to moderate inflammatory activation in the periphery (Suman et al., 2022; Allen et al., 2023; Wang Y. et al., 2023). Two Meta-analyses that included 17 (Feng L. et al., 2023) and 14 (Long et al., 2023) clinical cohort studies, respectively, reported that high levels of peripheral inflammation (blood CRP, TNF-α, and interleukin-6 (IL-6)) significantly increased the risk of cognitive decline in the elderly population (OR = 1.14; 95% CI: 1.03–1.27; *p* < 0.00001); and higher CRP levels were associated with an increased risk of dementia conversion (hazard ratio (HR) = 1.473; 95% CI: 1.037–2.090; *p* = 0.0394). Whereas populations with cognitive impairment also exhibit features of peripheral inflammatory activation, data from a cohort study of 2,479 patients ≥60 years of age in the United States showed that white blood cell counts (WBC), neutrophil counts, neutrophil-to-lymphocyte ratios, and neutrophil-to-albumin ratios in the peripheral blood of populations with cognitive impairment were significantly higher than those of normal populations (Li W. et al., 2023). AD, the most prevalent neurodegenerative disease, has been reported to have enhanced genotypic effects of the proteins SPI1 and CD33 encoding proteins at the AD inflammation-related SNP locus as peripheral blood CRP levels increase, and an increased risk of conversion to AD in people with mild cognitive impairment (MCI) (Huang J. et al., 2022). One study (Lyu et al., 2023) demonstrated that by comparing diabetic (T2DM) patients aged 29–65 years who did not meet the criteria for a clinical diagnosis of cognitive impairment with the normal population (42.125 ± 10.436 years), although the difference did not gain statistical significance, the Montreal cognitive assessment scale (MoCA) scores of the T2DM patients showed a tendency to be lower than that of healthy controls and peripheral blood laboratory results showed significantly lower levels of peripheral blood IL-4 and brain-derived neurotrophic factor (BDNF) in the T2DM population compared to the healthy population. Suggesting that peripheral blood cytokines and BDNF may have been altered in T2DM patients before the onset of cognitive deficits. Also, surface-based morphometry analysis of the differential brain areas in the two groups showed that the transverse frontal pole gyrus and sulcus depth of the left brain were positively correlated with peripheral blood levels of anti-inflammatory mediators, IL-10 (Spearman’s correlation, *R* = 0.636, *p* = 0.011). Furthermore, in addition to T2DM, clinical and epidemiological evidence suggests that specific diseases such as silicosis (Suman et al., 2022), chronic renal insufficiency (Wang H. et al., 2022; Wang Y. et al., 2023), and inflammation bowel disease (IBD) (Hopkins et al., 2021) are also associated with long-term cognitive impairment; for example, multivariate logistic regression analysis found that increased urinary β2-microglobulin levels were independently associated with cognitive deterioration in CKD patients (Wang Y. et al., 2023).

## 3 Affected brain regions and functional networks

**Figure 1:**
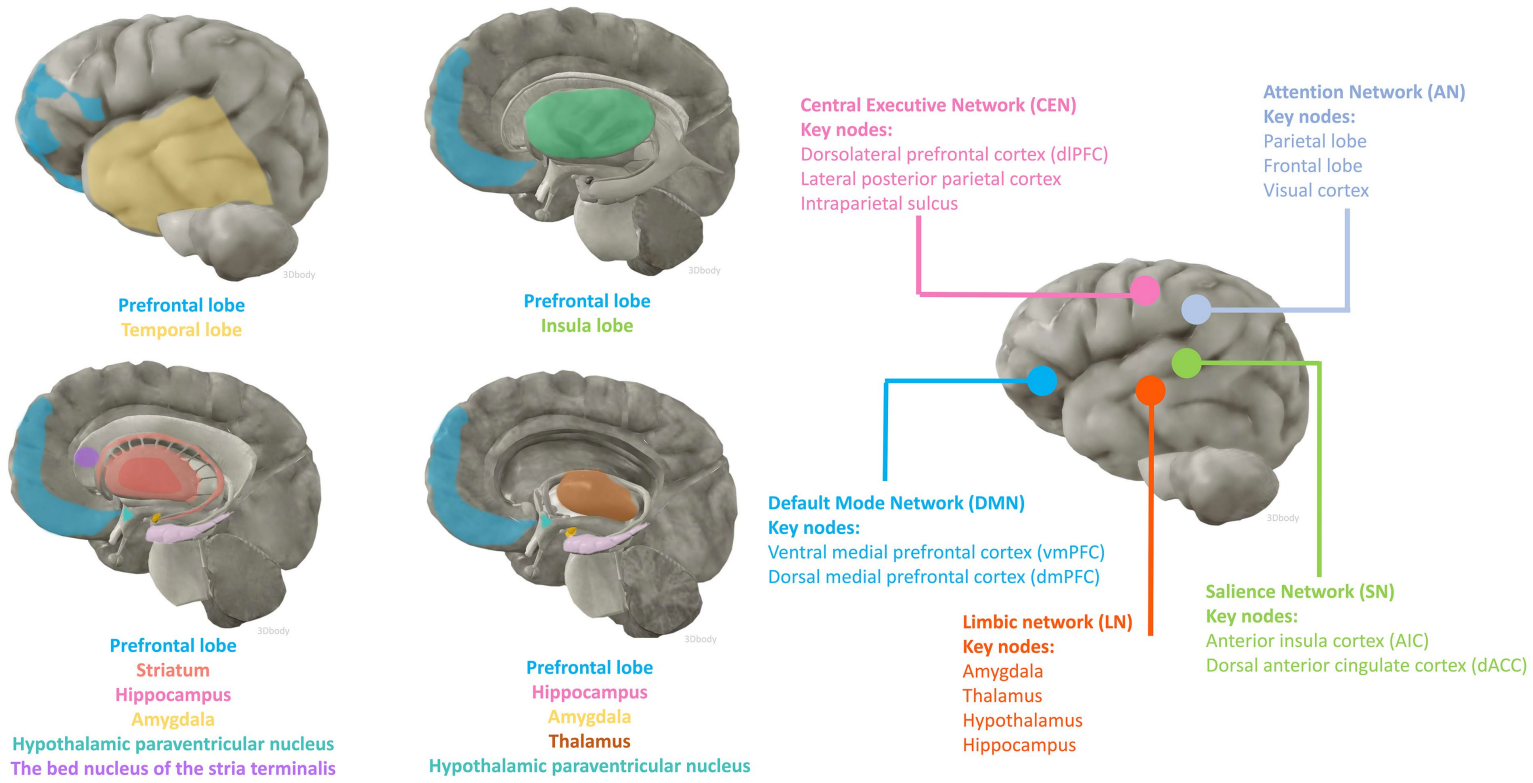
Brain regions and functional networks that may be affected by inflammation. The anatomical illustration of the brain was generated by 3Dbody software.

fMRI and fludeoxyglucose positron emission tomography are two of the most widely used non-invasive functional brain imaging techniques, which to some extent reflect the intracerebral network of functional connectivity associated with cognitive activity. Numerous animal studies have identified candidate brain regions involved in peripheral inflammation to perceive afferent visceral signals and initiate efferent neuroendocrine and autonomic responses. The main areas include the prefrontal, insula, and temporal cortex; the hippocampus; the striatum; the paraventricular nucleus of the hypothalamus, the amygdala, the bed nucleus of the stria terminalis (BNST), and the thalamus (Kraynak et al., 2018; Jagot et al., 2023). Anatomically, the amygdala and striatum make up the basal ganglia, and the limbic system consists of the limbic lobe and its closely associated subcortical structures such as the amygdala, BNST, the hypothalamus, and the anterior nucleus of the dorsal thalamus, suggesting an important role for the involvement of the cerebral cortex, the basal ganglia, and the limbic system in the perception and processing of peripheral inflammatory signals (Figure 1).

Large-scale functional networks consist of anatomically and functionally connected brain regions that show coherent activity by neuroimaging. The study of functional brain networks has not only yielded extensive descriptions of normal cognitive processes but has also contributed to the understanding of the neural underpinnings of neurocognitive impairment processes. Explaining brain function relies on the multi-scale properties inherent in brain anatomy, ranging from the synaptic connections of individual cells to organized assemblies of cellular regions, and from the connectivity of functional nuclei to the large-scale structure of interconnected brain regions. The most studied networks include the Default Mode Network (DMN), the Salience Network (SN), the Central Executive Network (CEN), and the Attentional Network (AN) as well as other networks including the Limbic Network (LN). In a cognitively normal population with a mean age of 65 years, higher peripheral levels of soluble TNF receptor-1 (TNFR1) and IL-6 were associated with reduced connectivity function in the DMN, a trend and correlation that was similarly validated in a cohort of people who progressed to MCI (Walker et al., 2020). Functional abnormalities in the DMN can be similarly observed in previous studies of AD pathologic progression and clinical manifestations of cognitive abnormalities (Chhatwal et al., 2018). Similarly, in cognitively unimpaired older populations (82–95 years), peripheral blood levels of IL-6 have been shown to correlate with a heavier whole-brain burden of extensive amyloid-β (Aβ) deposition and atrophy of the hippocampus, and high levels of peripheral proinflammatory factors may mediate deficits in verbal memory performance (Oberlin et al., 2021). This evidence suggests a phenomenon whereby peripheral inflammation can lead to abnormalities in functional connectivity between brain regions and certain pathologic changes associated with cognitive degenerative disorders before cognitive impairment is manifested. These alterations promote the progression and occurrence of future neurocognitive impairment.

We next explore the relationship between inflammation-involved regions and cognition based on various large-scale brain functional networks. The DMN has been associated with spontaneous cognitive function since its discovery in resting-state experiments (Wang M. et al., 2022; Wu et al., 2023). The DMN is divided into three subsystems: (i) ventral medial prefrontal cortex (vmPFC) and dorsomedial prefrontal cortex (dmPFC); (ii) posterior cingulate cortex (PCC) and medial precuneus (MPC); and (iii) a subsystem consisting of the lateral parietal cortex (LPC) (Wu et al., 2023). The subsystem comprising the prefrontal cortex (PFC) illustrates the dynamic homeostatic role played by the DMN, as it has been shown to connect areas responsible for the processing of exogenous sensory information (e.g., peripheral immune-inflammatory alterations) to the hypothalamus and the amygdala (Yegla and Foster, 2019; Mittli et al., 2023; Weng et al., 2023). Higher cognitive and executive functions in the PFC are preferentially affected in neuroinflammatory disorders, leading to executive dysfunction and severe neurocognitive impairments such as delirium (Arnsten et al., 2023). Inflammatory molecular dysregulation has been associated with reduced gray matter and or total volume in brain regions such as prefrontal, amygdala, and striatum (Savitz et al., 2015; Opel et al., 2019). fMRI findings reflect that peripheral inflammation levels are associated with decreased functional connectivity of the amygdala-PFC circuitry as well as impaired hippocampal function (Arnsten et al., 2023; Ge et al., 2023). Based on this evidence we hypothesized that the DMN network may be involved and affected in the processing of peripheral inflammatory and immune signals and that dysfunction of this network may contribute to the development of neurocognitive impairment.

The amygdala and striatum are two key regions that support emotional assessment and learning, and their altered developmental trajectories and functional connectivity have important implications for cognitive and behavioral development (Mitchell et al., 2014; O'Callaghan et al., 2014; Sharvin et al., 2023). While the striatum has been shown to be more associated with motor dysfunction in previous studies, there is growing evidence from functional neuroimaging studies in healthy individuals that striatal regions modulate behavior and cognition due to the strong anatomical connections of the striatum with many cortical regions including the frontal, temporal, and insula, as well as some subcortical regions (amygdala and hippocampus) (O'Callaghan et al., 2014). The amygdala is a core structure of the anterior medial temporal lobe and plays an important role in a variety of brain functions involving memory, emotion, perception, social cognition, and even consciousness (Dominguez-Borras and Vuilleumier, 2022). Altered functional connectivity associated with peripheral blood levels of inflammatory mediators has been observed in the brain of a community population of adolescents as young as 12–15 years of age, with higher blood levels of TNF-α associated with diminished connectivity between the right inferior frontal gyrus and the left parietal cortex, as well as altered connectivity between the right amygdala and the left striatum (Swartz et al., 2021). Evidence that higher levels of peripheral inflammation may exhibit reduced cortico-cortical connectivity as well as amygdala-parieto-occipital connectivity, leading to reduced emotion regulation and behavioral control, has been provided in other studies (Nusslock et al., 2019). In addition, functional neuroimaging studies in the treatment of hepatitis C or melanoma have shown that organismal fatigue due to interferon-alpha (IFN-α) treatment is mediated through altered striatal function (Capuron et al., 2012). In animal studies, upregulation of TNF-a expression and dysregulation of excitatory as well as inhibitory signaling in the basolateral amygdala can be induced by inducing chronic pain in the periphery, allowing for the emergence of an anxiety-like phenotype (Chen et al., 2013). Brain fMRI with behavioral assessment in rats at 6, 12, and 18 months, respectively, found that memory loss was associated with decreased functional connectivity within different memory systems, most notably alterations between the hippocampus and several regions including the striatum and amygdala (Febo et al., 2020). Co-regulation of insulin signaling between the hippocampus and central amygdala can affect synaptic function, behavior, and cognition (Soto et al., 2019). These correlations suggest that peripheral inflammation may affect functional connectivity in brain regions early in life and development.

Key nodes of the SN are located in the anterior insular cortex (AIC) and dorsal anterior cingulate cortex (dACC), whose functional connectivity has been shown to extend to the thalamus, hypothalamus, amygdala, substantia nigra/ventral pallidum, and temporal poles (Labrenz et al., 2019). The SN is activated in response to salient internal or external information in response to stimuli including alterations in the peripheral inflammatory environment, and its dorsal AIC (dAIC) is thought to play a major role in the regulating interactions between large-scale functional networks (Labrenz et al., 2019). Due to the significant mediating role of this network, dysfunctional synaptic processing leads to many diseases, including neurodegenerative disorders such as dementia (Chow et al., 2023). As previously described, the insular cortex is affected by alterations in peripheral immunity and regulates peripheral immunity through its ability to “immune-memory” (Koren et al., 2021). Similarly, aging, peripheral inflammation, and metabolic syndrome can induce a microinflammatory state within the hypothalamus, which further affects energy homeostasis and contributes to the progression of aging and metabolic disease (Tang et al., 2015). Activation of hypothalamus-enhanced adult neurons restores cognitive and emotional functions in AD (Li Y. D. et al., 2023). Whether these connections are involved in cognitive impairment due to peripheral immunity remains to be investigated. It also remains unclear whether other regions of the SN network are involved in this mechanism of peripheral and central interactions.

The CEN consists of a set of frontoparietal regions, mainly including the dorsolateral prefrontal cortex (dlPFC), the lateral posterior parietal cortex, and the intraparietal sulcus (Wang M. et al., 2022). The CEN has been associated with goal-directed behaviors and is involved in higher-order cognitive processes such as maintenance and manipulation of information in working memory, rule-based problem solving, and decision making (Meng et al., 2020). The dlPFC serves as a core region to perform working memory, recollection memory, abstract reasoning, and executive functions, and the ventral and medial regions (vmPFC) regulate emotional states, and the dlPFC and vmPFC are closely linked (Chen L. et al., 2023; Guo et al., 2023). It has been demonstrated that the selective α2-adrenoceptor (α2-AR) agonist guanfacine enhances dlPFC network connectivity and improves cognitive functions in the PFC by modulating cAMP-calcium signaling (Arnsten et al., 2023). Dexmedetomidine (Dex), also an α2-AR agonist, has been shown to have neurocognitive protective properties in a large number of studies, and whether it exerts its effects through a similar mechanism of action remains to be further investigated.

As one of the earliest brain networks we have recognized, the LN regulates many of the brain’s core functions, including response, reaction, behavior, emotion, memory, and learning (Quattrini et al., 2021). It generally includes the amygdala, thalamus, hypothalamus, and hippocampus, as well as many functionally and anatomically related regions that are considered paralimbic structures, including the prefrontal-limbic system, anterior cingulate cortex, medial temporal lobe network, para hippocampal gyrus, olfactory lobe, and ventral tegmental area (Onofrj et al., 2022). Considerable overlap of the limbic network with the DMN, SN, and CEN can be seen in the anatomical composition, and this overlap also reflects the functional associations between the networks (Jiang X. et al., 2023). This contains most of the inflammation-involved brain regions, thus explaining the peripheral inflammation-mediated impairment of memory and learning, both of which are often used as important dimensions in evaluating cognition.

The AN is involved in spontaneous, top-down allocation of attention. It consists primarily of the parietal and frontal lobes and visual cortex, but also includes subcortical structures such as the superior colliculus and thalamic occipital. Neuroimaging studies have shown that higher-order nodes (frontal and parietal cortex) are the source of generating attention-related signals that are further fed back to the visual cortex, in addition to the frontal–parietal lobes, which simultaneously function to maintain and control spatial attention (Fiebelkorn and Kastner, 2020). Since the brain AN is associated with most of the major cognitive processing, impairment of the AN can manifest as impaired cognitive processing (Onofrj et al., 2022). Higher levels of peripheral inflammation have been associated with lower AN connectivity in both depressed and AD elderly patients (Walker et al., 2020; Aruldass et al., 2021). In addition to attention and sensory deficits caused by neurodegenerative diseases, AN abnormality has been associated with neuropsychiatric disorders such as schizophrenia (Tosoni et al., 2023).

The functions of the brain are extremely complex. In addition to maintaining basic functions including consciousness, cognition, and rhythm by integrating functional networks, the brain also influences the peripheral immune status of the body. There are many research gaps regarding whether the central effects following peripheral activation of brain regions are positive or negative, such as the aforementioned ability of the brain to modulate peripheral immunity, which may inhibit or limit peripheral inflammatory responses. It is well known that the center regulates the immune response mainly through the autonomic and neuroendocrine systems (Koren et al., 2021). Less is known about the regulation of peripheral inflammation by different brain regions, nuclei, or functional networks. However, it is predictable that impairments in multiple functional regions and networks due to neuroinflammation can result in a dangerous skewing of the whole body involving various systems including impaired consciousness and cognition as well as impaired maintenance of immune homeostasis in the periphery. Peripheral inflammation may affect the brain before cognitive impairment is manifested, and there are differences in this effect across brain regions. This also flanks a causal situation in which peripheral inflammatory immune and inflammatory responses contribute to the onset of cognitive impairment by affecting functional changes in the brain.

## 4 Mechanisms by which peripheral inflammation promotes central inflammatory immune activation

### 4.1 Immunologic features of acute and chronic inflammation

The establishment of acute and chronic peripheral inflammatory stresses varies considerably in humans and model animals. Peripheral inflammatory events in humans tend to be acquired in naturally occurring situations, such as acute events, including surgery, trauma, and organ obstruction, and chronic events associated with aging and chronic disease. Infections can exist in two different situations, acute or chronic. In addition to aging, which is a naturally occurring condition, peripheral inflammatory events in model animals are often established in the context of human interventions, and commonly used methods include acute peripheral inflammation induced by limb fracture surgeries, open bowel physical stress, and injections of pro-inflammatory mediators, as well as by constructing models of chronic disease or inducing infections. We hypothesize that there are differences in the mechanisms leading to cognitive impairment acutely or chronically and that such differences may be mainly related to the type and level of cells and effector molecules involved in the inflammatory process, and that the reason for the differences may be due to the type of peripheral inflammation such as acute or chronic inflammation firstly, and secondly, may be related to differences in stress events such as infections caused by different pathogens, aseptic inflammation/injury, and different types of chronic diseases that ultimately lead to neurocognitive impairment through different mechanisms. In addition, there are also differences in the course of neurocognitive impairments, such as delirium and POCD, which often have an acute onset from strong stress exposures and chronic cognitive decline that manifests itself as a long-term, progressive exposure to low levels of inflammation (aging and chronic disease). In reality, strong inflammatory stress and chronic inflammation may coexist.

The normal aging process and coexisting chronic diseases allow for a peripheral and central shift to an overall pro-inflammatory environment, accompanied by increased levels of oxidative stress. This shift is often manifested by peripheral immune cells and central resident glial cells that are altered and in an activated state numerically, functionally, and phenotypically characterized (Lopez-Otin et al., 2023; Yuan et al., 2023). For example, senescent cells predominantly exhibit G1 or G2/M phase arrest, but this arrest does not imply inhibition of cellular metabolic activity but rather exhibits senescence-associated secretory phenotypes, such as IL-6, chemokines, insulin-like growth factor, matrix metalloproteins, serine proteases, etc., which contribute to the clearance of senescent cells by constructing an inflammatory microenvironment and promoting senescence (Lopez-Otin et al., 2023). Natural aging can be characterized by a balance of pro- and anti-inflammatory mediators in the body, but disruption of the balance gives rise to pathological manifestations associated with aging. The accumulation of persistent chronic inflammation, errors, and injury leads to pro-inflammatory shifts in the BBB and central immune cells and the internal environment. However, aging and some chronic diseases are distinctly different in their pathophysiology and immune profile. For example, aging results in diminished neutrophil, phagocyte, and DC phagocytosis, reduced numbers and proliferation of NK cells, NKT cells, T cells, and B cells, and reduced antibody affinity of B cells (Dodig et al., 2019; Lopez-Otin et al., 2023). Compared to aging, chronic diseases (e.g., IBD and silicosis) are characterized by significantly different peripheral immune profiles, such as increased IL1β ^+^ DCs and monocytes, enrichment of γδ T-cell subsets, and up-regulated expression of HLA-DR, a marker of T-cell (CD3^+^, CD4^+^, and CD8^+^) activation (Brilland et al., 2019; Boland et al., 2020; Mitsialis et al., 2020). Acute inflammatory stress also differs from chronic peripheral inflammation in the initial phase, the former is often accompanied by short-term exacerbations and intense immune cell activation, aggregation, and inflammatory storm formation, often with highly activated macrophages, neutrophils, and mast cells mediating activation of innate intrinsic immunity, e.g., COVID-19 and surgically-induced aseptic injury (Joaquim et al., 2022; Bhuiyan et al., 2023). Some acute inflammatory events can be followed by transformation into chronic inflammatory processes, e.g., long COVID-19, with long-term neurocognitive effects (Joaquim et al., 2022). Unlike chronic inflammation, in acute inflammation, after the pathogen is removed or stress is blocked, damaged tissue is healed and the body’s inflammatory status and immunity returns to normal. In healthy populations that do not have an aging phenotype or are not affected by chronic disease, a single peripheral inflammatory stress rarely causes acute neurocognitive impairment, which is why neurocognitive impairment tends to occur at a higher rate in chronically ill and elderly populations. We therefore observe more often that adaptive changes to acute injury in the context of aging and chronic inflammation are characterized by rapid activation and expansion of the inflammatory immune response, initiated by microglia inducing neuroinflammation with dysfunction of brain-immune pathways, which leads to the development of neurocognitive injury. Looking at the immune profile of acute activation and exacerbation of chronic inflammation, it seems that widely activated immune cells are deleterious because these cells mediate a rapidly expanding immune-inflammatory response that promotes neuroinflammation, however, interestingly, but in some specific cases, more in the course of is chronicity, depletion of lymphocytes or a decrease in their levels is correlated with deterioration of cognition, typical examples such as the depletion or decrease in the levels of B lymphocyte depletion exacerbates intracranial Aβ burden (Feng W. et al., 2023). Moreover, in older adults diagnosed with small cell lung cancer, T lymphocyte counts are lower in the cognitively impaired population (Soria-Comes et al., 2020). Explaining these seemingly contradictory phenomena relies on a deeper and more specific understanding of the acute and chronic immune response and the mechanisms underlying acute and chronic neurocognitive impairment.

**Figure 2:**
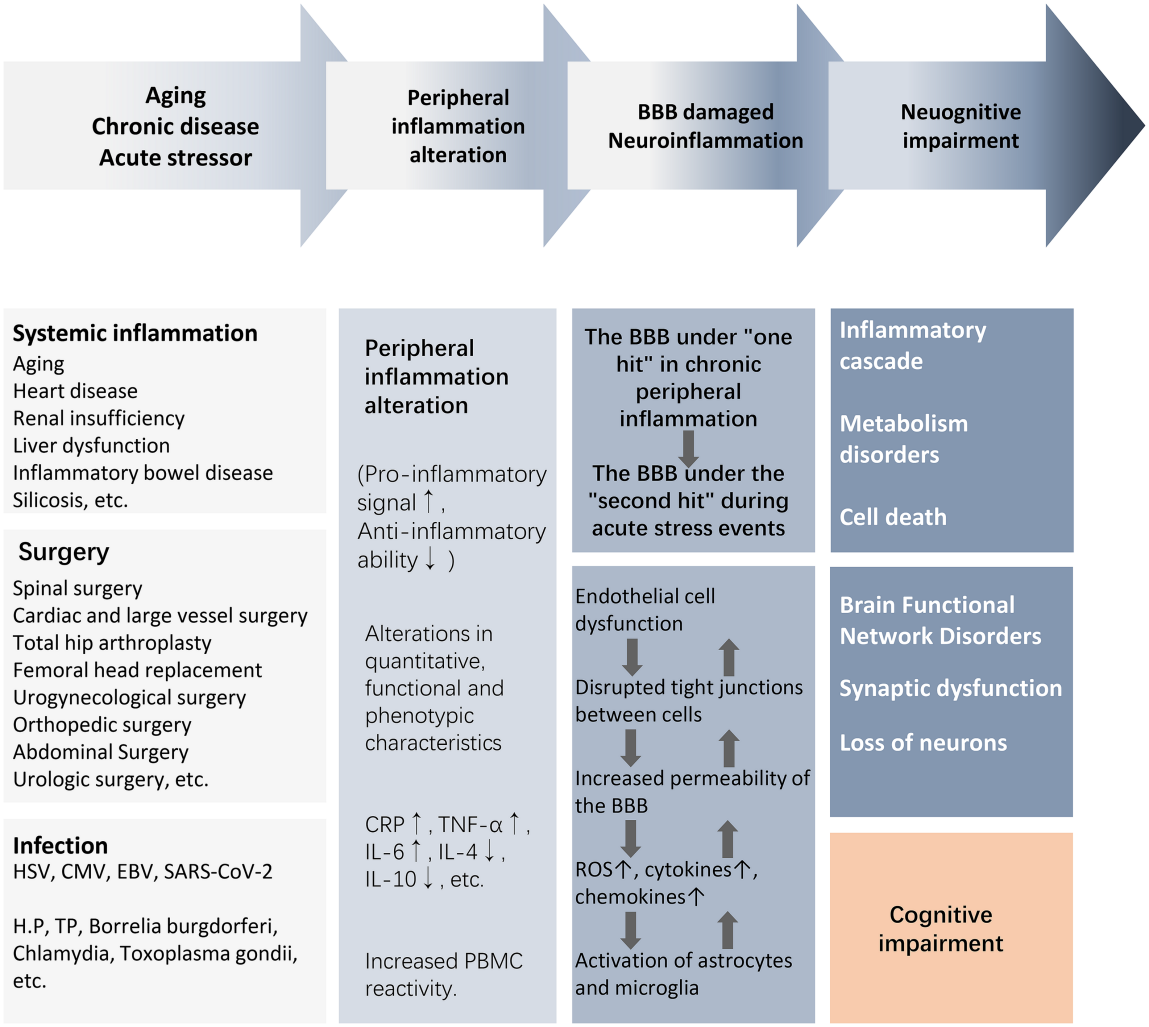
From peripheral inflammation to cognitive impairment. The activation of peripheral inflammation caused by surgery, infection, trauma, and chronic systemic inflammation can lead to the damage of the BBB and further mediate the occurrence of neuroinflammation, eventually causing neuronal energy metabolism and synaptic dysfunction, resulting in weakened brain functional connectivity and cognitive impairment. Abbreviations: BBB, blood–brain barrier; CMV, cytomegalovirus; CRP, C-reactive protein; EBV, Epstein–Barr virus; HSV, herpes simplex virus; IL, interleukin; NE, neutrophil count; NLR, neutrophil–lymphocyte ratio; NAR, neutrophil–albumin ratio; PBMC, peripheral blood mononuclear cells; ROS, reactive oxygen species; T2DM, type2 diabetes mellitus; TNF-α, tumor necrosis factor-α; WBC, white blood cell.

There is a lack of clarity regarding the core bridging mechanisms by which peripheral inflammation induces central neuroinflammation and cognitive impairment. For the elderly or chronically ill population experiencing or about to experience stressful events, mild blows may lead to severe consequences due to the increased vulnerability of the brain to external injury. For example, surgery, infection, and trauma cause upregulation of peripheral damage-associated molecular patterns (DAMPs), pathogen-associated molecular patterns (PAMPs), and cytokines, which recruit circulating immune cells to activate, while circulating inflammatory mediators and cytokines “second-strike” the BBB that has been damaged by chronic inflammation. In a short period of time, astrocytes and microglia are rapidly activated to release reactive oxygen species (ROS) and inflammatory cytokines, which further activate the peripheral/central inflammatory response, leading to endothelial cell dysfunction, disruption of intercellular tight junctions, and an increase in the permeability of the BBB, ultimately leading to synaptic dysfunction and neuronal loss (Figure 2) (Evered et al., 2022). As a result, acute and severe neurocognitive impairments can often be manifested in the immediate aftermath of a stressful event, such as delirium that occurs during awakening from anesthesia at the end of a surgical procedure (Evered et al., 2022). In older and chronically ill populations, the acute onset of neurocognitive impairment is not solely due to acute peripheral immune-inflammatory changes, but rather is the result of exacerbation of acute and chronic inflammatory activation.

### 4.2 Recruitment and infiltration of peripheral immune cells

Circulating immune cell profile alterations induced by peripheral injurious events are observed in both humans and model animals. Tissue and organ injuries associated with non-cranial surgery can cause significant whole blood cell alterations, such as significant upregulation of peripheral blood WBC and monocyte levels and platelet function activation after skin incision, characterized by systemic total CD14^+^ monocyte upregulation and CD4^+^ T cell activation (Hildenborg et al., 2022). In a peripheral trauma model constructed by amputation experiments on the distal (caudal) part of the zebrafish, leukocyte (mainly macrophage) invasion of the brain occurs during systemic inflammation, and on the first day after trauma, there is a significant upregulation of corpuscular protein 1A (coro1a)^+^ cell (macrophage, neutrophil, and lymphocyte) recruitment in the whole/midbrain and forebrain, and an increase in the mRNA levels of IL-1β, IL-34, IL-6, IL-8, and IL-10 mRNA expression were observed, and the injured zebrafish showed an increase in hyperactive (agitated) and avoidant behaviors (Chen et al., 2022). This finding supports a possible mechanism whereby peripheral inflammation condition alteration may affect brain function by promoting the infiltration of peripheral immune cells into the central nervous system (CNS).

**Figure 3:**
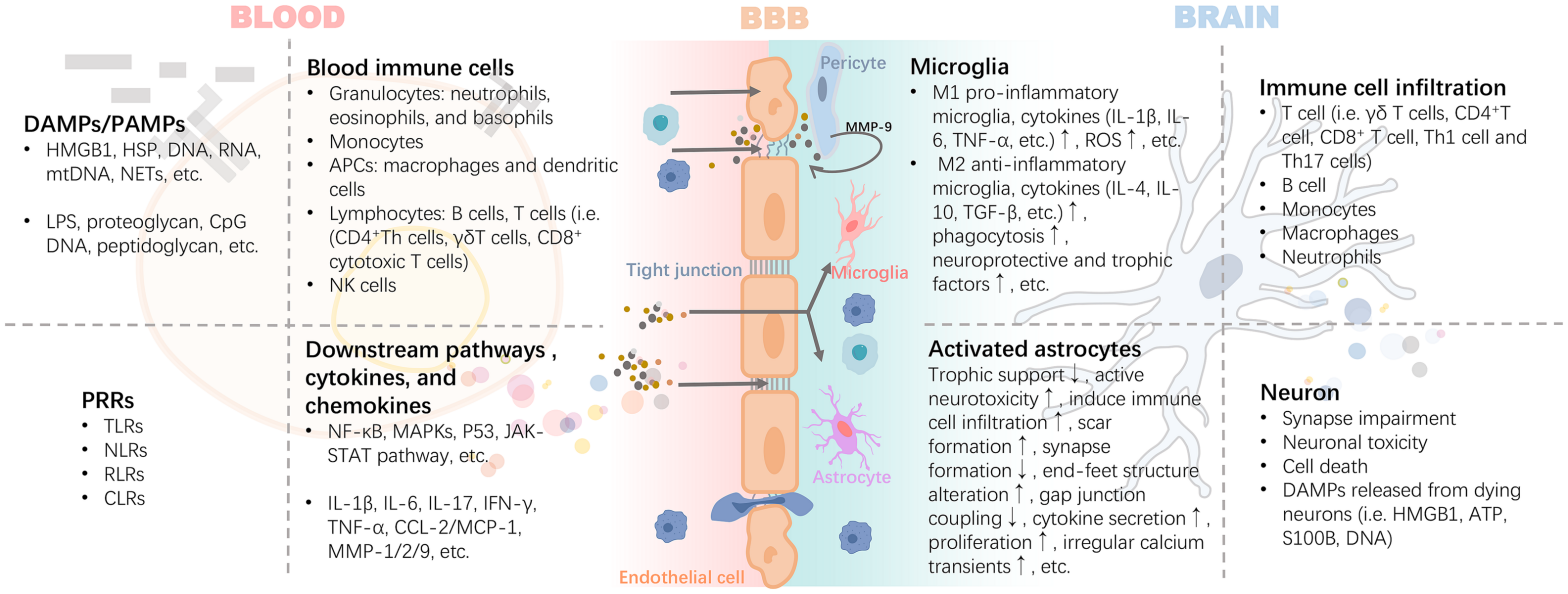
Inflammatory signals damage the BBB through blood circulation leading to neuroinflammation. The inflammatory cascade mediated by DAMPs/PAMPs-PRRs is the core precipitating mechanism of the inflammatory response induced by injury or infection. It includes the release of HMGB1 and HSP caused by injury, and proteoglycan caused by pathogen infection, which bind to PRRs, mediate the maturation or activation of immune cells through antigens, and release a variety of cytokines and chemokines. These pro-inflammatory mediators exert harmful effects on the BBB with blood circulation. Activated pericytes lead to the damage of BBB tight junction and further increase of permeability by secreting MMP-9. Infiltrating inflammatory factors or invasive peripheral inflammatory cells (i.e., B cell, T cell i.e., γδ T cells, CD4 + T cell, CD8+ T cell, Th1 cell, and Th17 cells) promote the polarization and pro-inflammatory phenotype of astrocytes and microglia. DAMPs released by the latter are further involved in the rapidly expanding inflammatory storm. Abbreviations: APCs, antigen-presenting cells; ATP, adenosine triphosphate; CCL2, chemokine (C-C motif) ligand 2; CLRs, c-type lectin receptors; CpG, cytidine-phosphatte-guanonine; DAMPs, damage-associated molecular patterns; HMGB1, high mobility group box 1; HSP, heat shock protein; IFN-γ, interferon-γ; IL, interleukin; MAPKs, mitogen-activated protein kinases; MCP-1, monocyte chemoattractant protein-1; MMP, matrix metalloproteinases; mtDNA, mitochondrial DNA; NETs, neutrophil extracellular traps; NF-κB, nuclear factor kappa B; NLRs, nucleotide oligomerization domain-like receptors; PAMPs, pathogen-associated molecular patterns; RLRs, retinoic acid-inducible gene-I like receptors; ROS, reactive oxygen species; S100B, S100 calcium-binding protein B; TGF-β, transforming growth factor-β; TLRs, toll-like receptors; TNF-α, tumor necrosis factor-α.

After activation of peripheral inflammation, immune cells in the blood can extensively invade and infiltrate brain tissue as well as the blood–brain barrier in response to chemokines. The infiltrating immune cells lead to the activation of glial cells and pericytes located around the BBB, accompanied by the activation of the NF-κB pathway and increased secretion of matrix metalloproteinases (e.g., MMP-9), which exacerbate the disruption of the tight junctions, thereby reinforcing the exchange of inflammatory signals from the periphery to the center (Takata et al., 2011; Qin et al., 2019) (Figure 3). For example, high levels of circulating mediators can cross the BBB and enter the brain parenchyma through the chemotactic effect of monocyte chemotactic protein 1 (MCP-1) on bone marrow-derived monocytes (BMDM) expressing the chemokine C-C motif receptor 2 (CCR2) (Naert and Rivest, 2012). In the presence of BMDM, resting microglia in the brain are activated to promote the release of pro-inflammatory cytokines, which disrupts the long-term potentiation (LTP) of synaptic plasticity required for learning and memory (Garre and Yang, 2018). A pro-inflammatory environment promotes the expression of CX3C chemokine ligand 1 (CX3CL1) and its receptor CX3CR1, as well as the chemokine (C-C motif) ligand 20 (CCL20), mediate immune cell chemotaxis. Also, the membrane-bound form of CX3CL1 acts as an adhesion molecule to mediate WBC capture and infiltration (Zhuang et al., 2019). In turn, CD4^+^CD28^+-^T cells infiltrate brain tissue driven by CX3CR1(Broux et al., 2012). CCL20 acts as a chemotactic agent for memory T cells and B lymphocytes to promote the recruitment of specific leukocyte subsets and T cells into the brain (Cabrera-Pastor et al., 2019). These signaling mechanisms may be involved in the invasion and infiltration of central immune-inflammatory cells resulting from peripheral inflammation.

### 4.3 The inflammatory cascade

**Figure 4:**
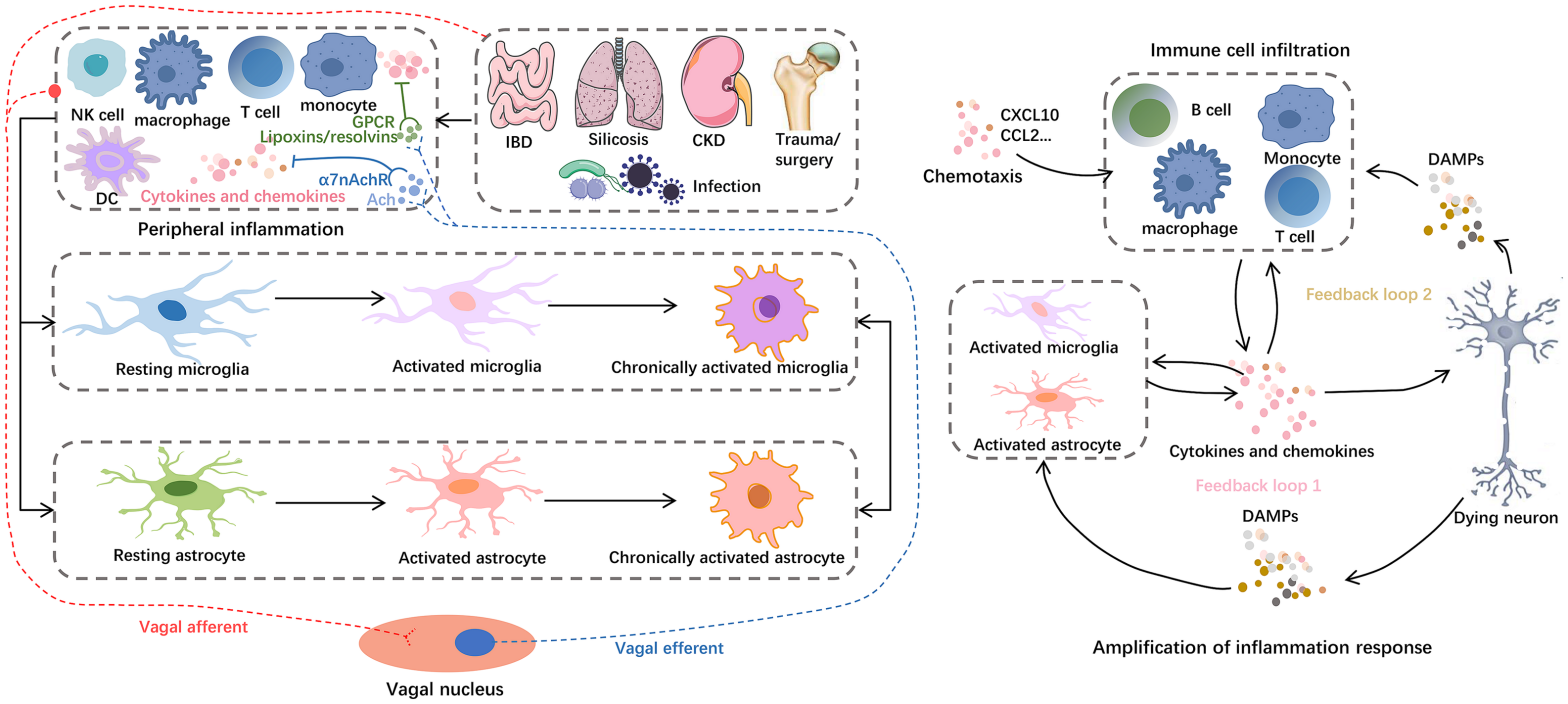
Inflammation storm centered on glial cells. For example, IBD, silicosis, CKD, hip fracture, and H.P. infection can cause peripheral central inflammatory activation, among which astrocytes and microglia play a central role in the progression of neuroinflammation. Glial cells reinforce and amplify the inflammatory cascade, from secreting chemokines to recruit peripheral immune cell infiltration to inducing neuronal cell death. On the one hand, peripheral inflammation can mediate the activation of central inflammatory signals through the vagal afferent pathway. On the other hand, the vagal efferent pathway can secrete ACh and lipoxins/resolvins to exert anti-inflammatory effects. These anti-inflammatory components inhibit the nuclear translocation of NF-κB in peripheral cells by binding to α7nAChRs and GPCRs, respectively. Abbreviations: Ach, acetylcholine; CKD, chronic kidney dysfunction; DAMPs, damage-associated molecular patterns; DC, dendritic cells; GPCRs, G protein–coupled receptors; IBD, inflammatory bowel disease; NKC, natural killer cells; α7nAchRs, α7 nicotinic acetylcholine receptors.

Despite the different contributing factors, the DAMPs/PAMPs-pattern recognition receptors (PRRs) axis activates downstream mechanisms that are thought to mediate important pathways in peripheral-central inflammatory signaling cascades. For example, aseptic trauma during surgery causes an increase in endogenous ligands such as high-mobility group box 1 (HMGB1), or circulating lipopolysaccharide (LPS) in patients with endotoxemia, both of which bind to PRRs on circulating cells, such as Toll-like receptors (TLRs), receptor for advanced glycosylation end-products (RAGE), γ-interferon receptor (IFN-γR), and IL-1R, and induce formation of receptor dimers triggering PRRs signaling axis (Poh et al., 2022; Barreto Chang et al., 2023a) (Figure 3). Recent studies (Squillace and Salvemini, 2022) have shown that TLRs involved in peripheral and central inflammation promoted by the physiological process of aging, as well as concomitant neurocognitively impaired disorders including cognitive deficits due to AD, craniofacial trauma, surgery, alcohol consumption, and infections/sepsis, etc. TLRs form homodimers and heterodimers through activation and activate several downstream signaling networks, including nuclear transcription factor-κB (NF-κB), mitogen-activated protein kinase (MAPK), P53 and JAK–STAT pathways through myeloid differentiation factor 88 (MyD88)-dependent and non-dependent pathways (Fann et al., 2018). Inflammatory signals promote an inflammatory response centered on astrocytes and microglia. Activated glial cells, together with other immune cells infiltrated in tissues, lead to neuronal dysfunction and death (Figure 4). The NF-κB pathway is a central mechanism involved in neuroinflammation and degeneration and can be activated by cytokines secreted by immune cells infiltrated in the blood and tissues such as TNF-α and IL-17. Inflammatory factors produced downstream of the pathway (including IL-1β, IL-6, COX2, CCL2, and CXCL10) recruit neutrophils and macrophages to participate in the process, triggering an inflammatory immune cascade from peripheral to central. At the same time, precursors IL-1β and IL-18 and the gene expression of inflammasome components including junction proteins, effector proteins, and related receptors are increased in the cytoplasm, forming inflammasome complexes by further recruitment of adaptor protein (apoptosis-associated speck-like protein containing a CARD, ASC) and cysteine aspartate protease (Caspase)-1/8. Thus, induces activation of cleaved Caspase-1/8 formation and promotes the cleavage of gephyrin D (GSDMD)-NT by GSDMD-FL to initiate pyroptosis (Poh et al., 2022). Activated Caspase-1 and -8 trigger apoptosis by cleaving total Caspase-3 to initiate another form of cell death, secondary necrosis, by cleaving GSDME-FL to GSDME-NT (Feng et al., 2018). In addition, although infections, such as SARS CoV-2 and CMV, can affect the CNS in several ways, such as direct viral invasion of the brain or leading to ischemic stroke induced by hemorrhagic coagulation abnormalities, severe systemic inflammation due to infections can cause neurological damage through the overproduction of inflammatory mediators, including complement factors, prostaglandins, cytokines, and chemokines (Leng et al., 2023).

### 4.4 Activation of resident immune cells

Peripheral inflammation leads to activation of centrally resident immune cells and impaired cognition. Peripheral injury induces activation of brain microglia and mast cells (including parenchymal and meningeal mast cells) and induces increased levels of inflammatory factors in the hypothalamus and hippocampus, decreased expression of hippocampal postsynaptic densin 95, and tight junction protein (Occludin) (Yang J. et al., 2022). Sepsis induces activation of the pro-inflammatory environment and cognitive impairment in the zebrafish brain (Nie et al., 2022), and prolonged intermittent LPS stimulation induces learning-memory dysfunction and elevated IL-6 expression in the frontal cortex of the brain as well as hippocampal tissues and a tendency to exhibit impaired learning in mice (Engler-Chiurazzi et al., 2023). As well as silicosis model mice showed both lung inflammation and upregulation of pro-inflammatory cytokines in the mouse hippocampus, synaptic damage, Aβ accumulation, and memory impairment (Suman et al., 2022).

**Figure 5:**
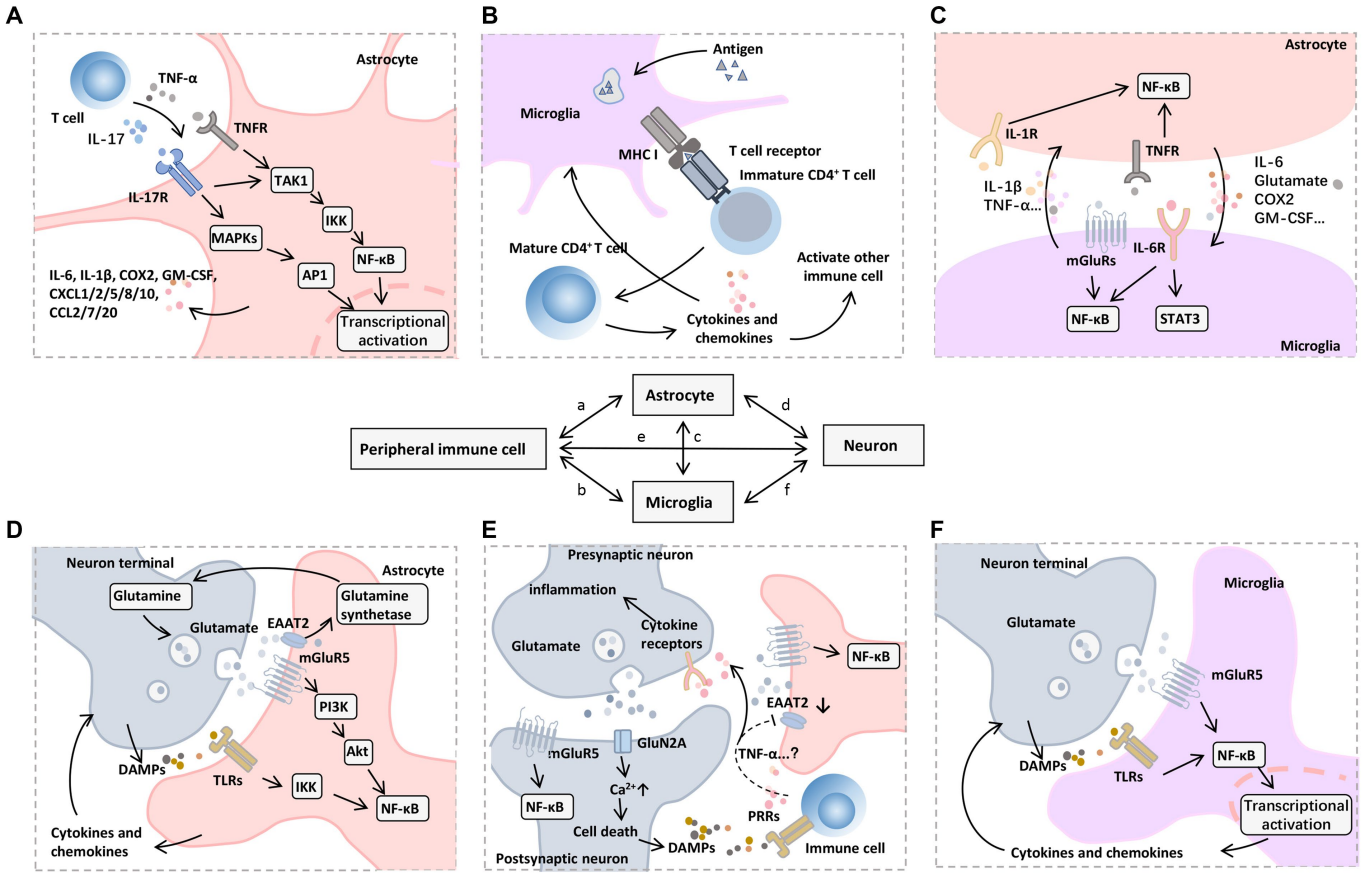
Mechanisms of neuroinflammation induced by peripheral immune cells. A variety of signaling pathways and transcription factors regulate neuroinflammatory responses, among which NF-κB is a central regulator of inflammation and neurodegeneration. **(A)** In astrocytes, NF-κB signaling can be triggered by proinflammatory mediators released by infiltrating immune cells, such as TNF-α and IL-17, which further promote TAK1 phosphorylation, activate MAPKs and IKKs, and cause a series of downstream proinflammatory (e.g., IL-6 and COX2) and chemotactic factors (e.g., CXCL1, CXCL2, CXCL5, CXCL8, CXCL10, CCL2, CCL7, and CCL20), which recruit other types of immune cells to participate in the inflammatory process. **(B)** Microglia as resident APCs can promote immune cell maturation through an antigen-presenting role, including CD4 + T cell activation mediated by T-cell receptor binding to MHC I. **(C)** Activated astrocytes and microglia interact with each other through the synthesis and release of proinflammatory mediators such as IL-1β, IL-6, and TNF-α to promote nuclear translocation of NF-κB, accompanied by the activation of JAK-STAT3 pathway. **(D)** Glutamate and its receptors also mediate the transmission of inflammatory signals. On the one hand, mGluR5 mediates glutamate to promote the nuclear translocation of NF-κB in postsynaptic and astrocytes through the PI3K-Akt pathway. Meanwhile, the glutamate transporter EAAT2 on astrocytes uptakes the free glutamate in the synaptic cleft into the cell and then promotes the synthesis of glutamine under glutamine synthetase, which can be further released to the extracellular and transported to the cytoplasm of presynaptic nerve terminals to produce glutamate by glutamine dehydrogenase. **(E)** On the other hand, cytokines (e.g., TNF-α) produced and released by immune cells or glial cells may down-regulate EAAT2 expression, leading to impaired glutamate uptake in the synaptic cleft. The accumulated glutamate can further bind mGluR5 and GluN2A receptors, which leads to excessive excitotoxicity of postsynaptic neurons and increased cytosolic Ca2+ levels, thus inducing cell death. **(F)** DAMPs released by dying neurons can further bind to PRRs on infiltrating immune cells and microglia, forming a vicious cycle of inflammatory response. Abbreviations: Akt, protein kinase B; APCs, antigen-presenting cells; COX, cyclooxygenase; CXCL, (C-X-C motif) ligand; DAMPs, damage-associated molecular patterns; EAAT2, excitatory amino acid transporter 2; GM-CSF, granulocyte-macrophage colony-stimulating factor; IKKs, IκB Kinase; IL, interleukin; JAK, Janus kinase; MAPKs, mitogen-activated protein kinases; mGluR5, metabotropic glutamate receptor type 5; MHC, major histocompatibility complex; NF-κB, nuclear factor kappa B; PI3K, phosphoinositide 3-kinase; STAT3, signal transducer and activator of transcription 3; TAK1, transforming growth factor β-activated kinase 1; TLRs, toll-like receptors; TNFR, TNF receptor; TNF-α, tumor necrosis factor-α.

Several central afferent pathways of locally or peripherally generated pro-inflammatory signals, including circulating pro-inflammatory cytokines through the compromised BBB (Figure 3) and vagal afferent pathways (Figure 4), facilitate the exchange of activated immune cells and cytokines between the peripheral immune system and the brain (Morris et al., 2015). These pathways culminate in the activation of astrocytes and microglia in the CNS and the release of a variety of pro-inflammatory molecules, including cytokines, chemokines, ROS, and nitric oxide, via peripheral pro-inflammatory mediators to promote a local immune response and lead to neuroinflammation (Ransohoff and Brown, 2012). Activated microglia can promote immune cell maturation through antigen presentation, and the latter secrete cytokines to promote glial cell activation, creating positive feedback of the immune response (Poppell et al., 2023). Glutamate-glutamine cycling and metabolic homeostasis can influence cell interactions, inflammatory signaling activation, modulation of cellular excitability, and cell survival (Zhang et al., 2022). As the most important excitatory neurotransmitter in the brain, glutamate binds to ionotropic receptors (e.g., N-methyl-D aspartate receptors (NMDAR) and non-NMDA receptors) and metabotropic glutamate receptors (mGluRs) (Zhang et al., 2022; Moller, 2023). The former mediates the process of rapid excitatory synaptic transmission through the formation of receptor-channel complexes, while the latter exerts its biological effects through the activation of coupled G-proteins to generate second messengers (Chakraborty et al., 2023). A robust literature supports the role of metabotropic glutamate receptor type 5 (mGluR5) and ionotropic receptors in the pathophysiology of cognitive impairment (Sitcheran et al., 2005; Shah et al., 2012; Ceprian and Fulton, 2019; Chen Y. et al., 2023). Ligand-bound mGluR5 activates transcription by promoting NF-κB nuclear translocation through the PI3K-Akt axis, causing the release of large amounts of proinflammatory mediators (Shah et al., 2012; Chen Y. et al., 2023). In addition, TNF-α may down-regulate the expression of glutamate transporter (EAAT2) (Sitcheran et al., 2005), which is mainly located on astrocytes, causing impaired glutamate transport in the synaptic gap, large amounts of stagnant glutamate in the gap binding to NMDAR, and repeated opening of Ca^2+^ leading to lethal Ca^2+^ overload. However, whether other cytokines affect EAAT2 expression is unclear (Figure 5). In addition, glutamate binding to non-NMDARs (e.g., d-amino-3-hydroxy-5-methyl-4-isoxapurine, AMPA) leads to the opening of Na^+^ channels, and a large amount of intracellularly transferred Na^+^ can cause acute cell swelling, resulting in neuronal death (Ceprian and Fulton, 2019). These mechanisms may explain the intrinsic cause of the delirium that occurs during the patient’s awakening from major surgery and for 1–7 days postoperatively.

## 5 Prevention and treatment strategies based on peripheral inflammatory immune regulation

### 5.1 Holistic-based anti-inflammatory strategies

Drugs, peptides, neutralizing antibodies, and blood products with immunomodulatory or anti-inflammatory potential may play a beneficial role in promoting inflammatory immune homeostasis in the body. In addition to intervening and removing factors that induce peripheral inflammation, drugs such as ulinastatin (Kim et al., 2023), α2-AR agonists Dex (Oxlund et al., 2022) and guanfacine (Arnsten et al., 2023), cyclooxygenase (Cox) inhibitors (Weng et al., 2021), and lidocaine (Geng et al., 2023) all exert anti-inflammatory or neuroprotective effects to attenuate cognitive impairment through various pathways. Peptides consisting of arginine, valine, leucine, and phenylalanine (Li T. et al., 2023) and proline-rich peptides (Banasiak-Cieslar et al., 2023), as well as neutralizing antibodies, such as anti-IL-17 (Cristiano et al., 2019) and anti-IL-6 (Hisaoka-Nakashima et al., 2022), have also been shown to have a positive effect in limiting inflammation and thus improving cognition.

In addition to the above, exercise and physical activity are thought to promote inflammatory homeostasis in the body (Ding and Xu, 2023). Extensive research has linked regular physical activity to an overall enhancement of cognitive function across the lifespan, but observations in human trials have not yielded consistent results. A Meta-analysis supported the evidence that physical activity is associated with a lower incidence of all-cause dementia and AD (Iso-Markku et al., 2022). However, in a recent RCT, aerobic, strength, and functional exercise did not lead to improvements in situational memory or executive function in older adults (Lenze et al., 2022). Also, the combination of cognitive rehabilitation and exercise did not appear to improve cognitive processing speed in patients with progressive multiple sclerosis (Feinstein et al., 2023). Conclusions from a review of multiple RCTs suggest that the link between regular physical activity and cognitive benefits in healthy populations is unknown (Ciria et al., 2023). Findings from animal studies reflect a positive picture. “Runner’s plasma” collected from mice that voluntarily ran injected into non-exercising mice reduced baseline neuroinflammatory gene expression and LPS-induced peripheral and cerebral inflammation, and that clusterin protein (a heterodimeric sulfated glycoprotein) may mediate this protective effect [90]. In addition to “exercise plasma,” plasma from young individuals was found to improve neuroinflammation and cognitive impairment in the hippocampus of aged mice (Schroer et al., 2023).

Plasma therapy may be beneficial. An international multicenter clinical study found that plasma exchange combined with immunoglobulin and albumin therapy significantly improved cognition and memory, language, processing speed, and quality of life in patients with mild-to-moderate AD without causing deterioration in their psycho-emotional status (Boada et al., 2020). After treating adult female mice with middle cerebral artery embolism by injecting plasma from healthy control, it was found that healthy plasma treatment promoted the recovery of neuromotor function after stroke and reduced infarct volume, cerebral edema, and brain atrophy (Mamtilahun et al., 2021). Also, Immunofluorescence staining showed a reduction in the number of apoptotic neuronal cells, degradation of tight junction proteins, and leakage of IgG in the infarcted marginal zone of the brain tissue of plasma-injected stroke mice compared with controls, indicating a correlation between the peripheral circulatory environment and neurocognitive function or the structural and functional integrity of the BBB (Mamtilahun et al., 2021).

### 5.2 Confusion faced in practice

There are still some conflicting results here regarding the drugs available, such as propofol, Dex, and Cox inhibitors. We have used all three of these classes of medications as prophylaxis and treatment of acute neurocognitive impairment in clinical practice, and in our previous experience, similar to results reported in the literature, these medications have not demonstrated consistent and stable efficacy. Propofol, a γ-aminobutyric acid type A receptor (GABA_A_R) agonist, a commonly used intravenous anesthetic for surgical anesthesia, has recently been shown to have potential anti-inflammatory capabilities (Zhang B. et al., 2021). However, the use of propofol often faces a dilemma of choice when considering the risk–benefit in elderly surgical or high-risk patients, as contradictory effects of propofol on either protective (Li et al., 2022) or injurious (Sun et al., 2019) cognitive function have been observed in clinical practice. Research on the activation/inhibition of GABA signaling on inflammation or cognitive shifts also remains controversial. Mincheva et al. reported that Golexanolone, a GABA_A_R antagonist, restored motor coordination, and spatial and short-term memory in hyperammonemia rats by reversing changes in peripheral inflammation, microglia, and astrocyte activation through decreased GABA_A_R activation (Mincheva et al., 2022). Surgery-induced upregulation of hippocampal NLR family pyrin domain-containing 3 (NLRP3) and increased GABA synthesis in aged mice resulted in neuroinflammation and cognitive deficits (Wang L.Y. et al., 2023). However, other studies have observed a causal relationship between reduced GABA levels and cognitive deficits, such as executive dysfunction in the medial prefrontal cortex (Yang Y. et al., 2022) and cingulate cortex (Fu et al., 2023). Contributing to this discrepancy may be related to the fact that GABA_A_R activation has significant diversity and complexity effects on synaptic function and plasticity (Koh et al., 2023). The specific mechanisms by which propofol exerts neurocognitive protective effects via peripheral and or central need to be supported by more research evidence. Considering that excessive propofol may cause unfavorable cardiovascular and hemodynamic adverse effects, whereas effective perfusion is essential for normal brain function, and that the elderly population is physiologically fragile, clinical decision-making should be informed by moderation, monitoring, and a good assessment of risk–benefit.

Dex is commonly used for sedation in patients undergoing surgical anesthesia and mechanical ventilation in the ICU, and in recent years Dex has come to the attention of researchers due to its anti-inflammatory/stress and neuroprotective potential. A random effects model-based study of 1,626 adults aged 60 years and older undergoing general anesthesia for surgical procedures showed that Dex significantly reduced the incidence of POCD (Yu et al., 2022). In the preclinical study, Dex reversed LPS-induced cognitive decline and promoted inflammatory regression (Li et al., 2020). However, it has also been demonstrated that there is no significant reduction in the incidence of postoperative cognitive impairment after Dex intervention (Deiner et al., 2017). Notably, Dex has excellent anti-inflammatory and stress-suppressing as well as sleep-promoting properties, and most studies support its neurocognitive protective potential.

Reductions in biologically relevant features of inflammation *in vivo* after Cox inhibitor administration in preclinical models also translate into cognitive protection. For example, the nonsteroidal anti-inflammatory drug ibuprofen was found to reverse peripheral/central inflammation and improve learning in an elderly model of cognitive impairment after laparotomy in mice (Huang et al., 2018). Dexketoprofen and etodolac reversed cognitive deficits in 3xTg-AD mice while reducing Aβ plaque deposition in the hippocampus (Pauls et al., 2021). A cross-sectional study including 1,866 subjects showed that oral aspirin was temporally associated with slower progression in cognitive decline compared to no aspirin application in a population with AD (Weng et al., 2021). Yet another study noted that in healthy older adults, low-dose aspirin did not significantly reduce the incidence of AD, MCI, or cognitive decline (Ryan et al., 2020). In addition, clinical applications should consider the adverse effects of bleeding and digestive tract damage, and in general, we need more evidence to support the pharmacological mechanisms of action of these drugs affecting neurocognitive function.

### 5.3 Strategies based on the peripheral organ-brain physiological axis

Accumulating evidence elucidates the physiological interactions linking peripheral organs to the brain. Potential adverse neurocognitive effects may be mitigated by intervening and modifying the pathophysiologic functions of the involved organs. The most studied of these is the microbial-gut-brain axis, in addition to the liver-brain axis, kidney-brain axis, lung-brain axis, and heart-brain axis.

Peripheral inflammatory events can lead to altered gut flora, cognitive impairments, and other brain dysfunctional disorders that exhibit different gut flora characteristics relative to normal. Gut flora affects normal brain development and behavioral function, and developmental exposure to microbial pathogens leads to behavioral abnormalities and cognitive impairment (Diaz Heijtz et al., 2011). Disruption of normal microbiota-gut-brain signaling may adversely affect the PFC, amygdala, hippocampus, hypothalamus, and striatum. In addition, neuroimaging studies have revealed the role of the microbiota in modulating the functional connectivity and structure of specific brain regions that can be traced to neurocognitive and behavioral outputs (Hoban et al., 2017; Caliskan et al., 2022; Kuijer and Steenbergen, 2023). Gut flora abundance and structural interventions may play a role in improving cognition by modulating peripheral inflammatory states. In human studies, additional administration of probiotic supplements or flora transplantation may benefit elderly population at high risk for POCD (Wang et al., 2021), cognitive impairment, and dementia (Murray et al., 2022; Park et al., 2022). In addition, healthier dietary patterns (e.g., appropriate cholesterol and fat intake, adequate amounts of dietary fiber and vitamins, and reduced unnecessary sugar intake) are accompanied by similar beneficial changes (Fu et al., 2022; Huang X. et al., 2022; Kim et al., 2022). In animal models, modification of gut bacteria through dietary interventions has shown promising results in terms of improved memory and reduced inflammation (Proctor et al., 2017; Kendig et al., 2023).

The liver, as an organ responsible for regulating metabolism and supporting the immune system, may be a key organ influencing cognitive development and prognosis. Recent studies have shown that the progression of chronic liver diseases, such as liver fibrosis and non-alcoholic fatty liver disease (NAFLD), is associated with cognitive decline and structural changes in the brain (Merli et al., 2013; Leone et al., 2023). Abnormal liver function may contribute to increased levels of peripheral inflammatory responses and decreased clearance of harmful substances such as Aβ (Wiest et al., 2021; Jiang R. et al., 2023). The mitigation of neurocognitive impairments may be exerted by treatment of the primary disease (e.g., NAFLD and hepatic fibrosis) or compensatory measures of liver function (e.g., albumin infusion) (Cheon and Song, 2022; Fagan et al., 2023). In addition, several lines of evidence have demonstrated beneficial effects on liver function and neurocognition by reestablishing intestinal flora homeostasis (Cheon and Song, 2022; Smith et al., 2023).

Cognitive deficits are prevalent in patients with different stages of CKD, and there is a significant correlation between CKD and AD pathologic features (Lidgard et al., 2022; Janelidze et al., 2023). The “kidney-brain axis” hypothesis emphasizes the role of kidney function in regulating neurodegeneration and cerebral blood flow. Interactions between the kidney and the brain may include the release of cytokines and chemokines, ROS production, and the circulation and localization of trophic factors and renin-angiotensin system (RAS) molecules (Ortiz et al., 2022; Tang et al., 2022). Proteinuria and glomerular filtration rate levels are considered important predictors of kidney-related neurocognitive impairment, as both reflect systemic vascular endothelial function to some extent (Scheppach et al., 2020; Lidgard et al., 2022). Understanding current therapies for renal disease is significant for associated neurocognitive impairment, such as recombinant human erythropoietin (rhEPO) (Shim et al., 2022) and RAS inhibitors (Wu et al., 2022), the former of which, as a key regulator of erythropoiesis, has been demonstrated to have immunomodulatory potential and to provide a beneficial role in preclinical models of neurocognitive impairment (Shim et al., 2022; Singh et al., 2023). However, the use of rhEPO is subject to adverse effects that may lead to excessive erythropoiesis, resulting in thrombosis (Eggold and Rankin, 2019). HMGB1 has recently been found to be significantly elevated in patients with CKD, promoting the disease feature of vascular fragility, and HMGB1 antagonists such as K883 have now been tested in preclinical therapeutic studies in CKD and neurodegenerative diseases (Paudel et al., 2020). Furthermore, considering the key role of oxidative stress in CKD-induced cognitive impairment, antioxidant therapy may be a promising option (Calabrese et al., 2020).

Chronic allergic lung inflammation or systemic inflammation due to chronic obstructive pulmonary disease (COPD) may adversely affect neurobehavior, and the evidence here supports the central nervous effects of peripheral inflammation (Pelgrim et al., 2019; Kanaya et al., 2022). The current definition of the “lung-brain axis” focuses more on the complex regulatory relationship between the microbiome, the lungs, and the brain, i.e., the microbiome located in the lungs can influence the both organs and participate in the regulation of respiratory and neurological diseases, and conversely, respiratory and neurological diseases can lead to alterations in the structure and diversity of the lung microbiome (Hosang et al., 2022). However, the pathways and specific mechanisms by which altered lung flora in pathological states affect CNS and cognition are unclear. In previous studies, modulation of the lung microbiota by neomycin or probiotic nebulization therapy was found to promote immune surveillance of lung metastases (Le Noci et al., 2018). In a recent study, pretreatment with neomycin tracheal perfusion resulted in localized changes in the lung microbiome such as increased abundance of *Mycobacterium avium* to modulate the activation state of central microglia (Hosang et al., 2022). We propose to envision whether reestablishing lung microbiota homeostasis through pharmacological intervention or mycobacterial transplantation could have a beneficial effect on treating the primary disease and combating neurocognitive impairment. Whether this effect is exerted by promoting immune homeostasis needs to be supported by more research evidence.

The heart-brain axis has been implicated in the onset and progression of dementia. Cognitive impairment is more common in patients with myocardial infarction and chronic heart failure, as the progression of these cardiac diseases often implies endothelial dysfunction and impaired microcirculation, the latter of which often mediates pathologic processes in the center (Jinawong et al., 2021; Komori et al., 2023). In a non-invasive optical pacemaker-constructed mouse model of tachycardia, an increase in heart rate activates the endogenous expression of Fos mRNA in CEN-related regions of the brain, which mediates the onset of anxiety (Hsueh et al., 2023). In addition, cardiac hypertrophy and heart failure models trigger macrophage infiltration and pineal neuronal loss in the mouse superior cervical ganglion, and neuronal numbers and peripheral melatonin levels in the pineal gland can be rescued by depletion of macrophages in the ganglion (Ziegler et al., 2023). These studies suggest a potential role for anti-inflammatory mechanisms that maintain normal cardiac physiology and drive heart-brain interactions in the potential modulation of brain function, providing research ideas for combating cardiac-associated neurocognitive injury. Overall, further exploration of the physiological interactions between peripheral organs or systems and the brain may provide strategies and ideas to “remotely modulate” brain function and prevent neurocognitive impairments.

## 6 Conclusion

This article reviews the correlation and mechanisms associated with peripheral inflammatory and neurocognitive impairment as well as anti-inflammatory prevention and treatment strategies. Studying the characteristics of peripheral inflammation associated with neurocognitive impairment and the mechanism leading to neuroinflammation helps develop early diagnosis, prognosis evaluation, and prevention methods based on peripheral blood circulation system sampling and intervention. Peripheral inflammatory responses due to stressful events such as surgery and infection can induce alterations in neuroinflammation features through multiple pathways, and inflammation-based strategies to intervene peripherally and or centrally may ameliorate impairments in neurological and cognitive function. Despite much progress, bridges across the mechanisms of action of peripheral-central inflammation remain to be further investigated.

## Author contributions

ST: Conceptualization, Data curation, Formal analysis, Software, Visualization, Writing – original draft, Writing – review & editing. WC: Conceptualization, Data curation, Visualization, Writing – review & editing. GK: Conceptualization, Data curation, Formal analysis. Visualization, Writing – review & editing. LW: Conceptualization, Data curation, Formal analysis, Funding acquisition, Software, Visualization, Writing – review & editing. YX: Conceptualization, Data curation, Formal analysis, Funding acquisition, Software, Visualization, Writing – review & editing.
